# *Who’smore vulnerable?* A generational investigation of COVID-19 perceptions’ effect on Organisational citizenship Behaviours in the MENA region: job insecurity, burnout and job satisfaction as mediators

**DOI:** 10.1186/s12889-021-11976-2

**Published:** 2021-10-27

**Authors:** Ali B. Mahmoud, Dieu Hack-Polay, William D. Reisel, Leonora Fuxman, Nicholas Grigoriou, Iris Mohr, Raneem Aizouk

**Affiliations:** 1 University of Wales Trinity Saint David, London, UK; 2 St. John’s University, New York, USA; 3 Crandall University, Moncton, Canada; 4 Monash University, Clayton, VIC Australia; 5 City, University of London, London, UK

**Keywords:** COVID-19 perception, Job insecurity, Burnout, Job satisfaction, Organisational citizenship behaviour (OCB), Generational differences, Multi-group analysis, Partial-least-square structural equation modelling, MENA

## Abstract

**Background:**

This paper is an empirical investigation that examines a path model linking COVID-19 perceptions to organisational citizenship behaviour (OCBs) via three mediators: job insecurity, burnout, and job satisfaction. The research examines the path model invariance spanning Generations X, Y, and Z. Three countries in the Middle East and North Africa (MENA) were the focus of the study.

**Methods:**

The data was collected from a sample of employees in service companies (*n* = 578). We used a Partial Least Square Structural Equation Modelling (PLS-SEM) to analyse the data.

**Results:**

Our findings reveal that COVID-19 perceptions positively predict job insecurity, which positively impacts burnout levels. Burnout negatively predicts job satisfaction. The findings established that job satisfaction positively predicts OCBs. The mediation analysis determined that job insecurity, burnout and job satisfaction convey the indirect effects of COVID-19 perceptions onto OCBs. Finally, our hypothesised model is non-equivalent across Generations X, Y and Z. In that regard, our multi-group analysis revealed that the indirect effects of COVID-19 perceptions on OCBs were only valid amongst younger generations, i.e., Generation Y and Generation Z. Specifically, younger generations are substantially more vulnerable to the indirect effects of COVID-19 perceptions on their engagement in OCBs than Generation X whose job satisfaction blocks the effects of COVID-19 perceptions on OCBs.

**Conclusions:**

The present study extends our knowledge of workplace generational differences in responding to the perceptions of crises or pandemics. It offers evidence that suggests that burnout, job attitudes and organisational outcomes change differently across generations in pandemic times.

**Supplementary Information:**

The online version contains supplementary material available at 10.1186/s12889-021-11976-2.

## Introduction

The causes and consequences of job insecurity have been the subject of substantial research in the last four decades since the publication of the seminal theory paper on job insecurity [1]. The reasons for this rising interest in job insecurity research is due to the breadth of its impact: (1) the severe negative outcomes for workers anxious about their employment; (2) the explicit deleterious organisational effects. A series of meta-analyses have discovered different categories of adverse effects of job insecurity on the health of employees, their attitudes and the level of stress they experience [see multiple meta-analytic studies: [2–6]. Job insecurity is a public health dilemma. For example, a systematic review [7] found that job insecurity and unemployment were strongly linked to mental health, whereas job insecurity correlated more firmly with somatic symptoms. The consequences of job insecurity affect individual attitudes and behaviours, raising negative implications for the organisation. When faced with job insecurity, employees’ performance deteriorates; they engage in counterproductive workplace behaviours, making fewer voluntary contributions thus impacting organisational citizenship behaviour [8], which is defined ‘as individual discretionary behaviour that is not directly or explicitly recognised by the formal reward system, and that in aggregate promotes the effective functioning of the organisation’ [9]. Sverke et al.’s meta-analysis concluded that job-insecure employees tend to be less likely to contribute to achieving the organisational strategy [6]. This fundamental inter-relationship between employees and employers means that negative effects have individual and organisational manifestations.

This research focuses on an aspect of job attitudes and organisational outcomes amid the COVID-19 pandemic. We seek to understand if COVID-19 influences employee perceptions of job insecurity and how this influences attitudes and behaviours. We ground this in both theory and evidence. We focus upon employees’ contextual performance to explore the degree to which generational differences influence discretionary organisational citizenship behaviours (OCBs). We first validate our hypothesised model to learn how COVID-19 perceptions predict OCBs via job insecurity, given that employees have few safeguards against macro forces such as global GDP declines resulting from the pandemic. Second, we examine whether generational differences (among Generations X, Y, and Z) can moderate the hypothesised model. This approach has not been previously reported in the literature. Our study took place in the MENA geographical region, where the most recent COVID-19-related research has focused on the health sciences [10]. MENA region, spanning 20 countries across the Middle East and North Africa (hence acronym MENA), is particularly vulnerable to increased unemployment due to the COVID-19, which stems from economic and financial hardships organisations are exposed to due to pandemic-induced contracting of business activities. Moreover, the MENA region has long suffered one of the highest rates of youth unemployment globally (30% as of 2017)— its young people are five times more at risk of unemployment than their counterparts in other regions [11]. Therefore, the MENA region offers an ideal context to study employees’ attitudinal and behavioural responses to COVID-19-triggered uncertainty, notably amongst younger generations, i.e., Generation Y and Generation Z compared to their ancestors.

Our study focuses on the psychological aspects of employment in the service sector, where the pandemic crisis has severely battered employment conditions worldwide. Such precarity has been attributed to the services sector employees’ limited ability to shift to the safety of remote or virtual work since their job nature requires them to interact with customers directly. Moreover, it impacts customer service delivery that is particularly vulnerable when employees who engage with customers directly perceive their job security as being threatened [12], making the services industry an excellent context for our investigation.

## Literature review and conceptual model

### COVID-19 perceptions

This paper seeks to examine the association between COVID-19 perceptions and job insecurity, representing one of the first research studies linking these two constructs and subsequent effects upon OCBs. We identified one prior study [13] that examined the relationship between job insecurity and safety compliance in the US during the pandemic. Our rationale for the association is grounded in job insecurity theory and evidence from the MENA region. From a theoretical standpoint, we know from an extensive body of research that employees experience increased job insecurity perceptions when they have greater uncertainty about their job status [14]. This can occur for structural reasons such as downsizing and strategic change, or it can occur from broad effects like macroeconomic shifts. Uncertainty is very stressful to employees, yet there is often little in the way of responses that employees can use to address the threat of job loss. COVID-19 perceptions precipitate this sense of angst and worry because COVID-19 has traversed the world leaving unparalleled threats to society and economic systems. In particular, nations in the MENA region have the added challenge of systemic challenges to an adequate COVID-19 response due to vaccine production taking place elsewhere, the substantial decline in demand in sectors such as oil, heavy reliance on service economies, and corruption among politicians [15]. Prior research into macro influences, such as war, has also been shown to predict job insecurity e.g. [16]. We reason that the scale of COVID-19 and its detrimental impacts upon GDP inside the MENA region will be positively related to job insecurity. We define COVID-19 perceptions as the perceived probability of discomfort and/or worry about the potential negative impact on an individual [17].

The COVID-19 pandemic has dramatically disrupted the global business environment and workplace practices. It is broadly acknowledged that COVID-19 has contributed to the wholesale decline of businesses in 2020 [18, 19]. Businesses that survived did so by reducing expenses, often targeting human capital reductions. These aggressive initiatives affect a variety of organisational domains and increase employee job insecurity. Moreover, the political and socio-economic landscape that businesses operate in and strategies adopted by firms have been tremendously redefined by COVID-19 [20, 21].

Given that COVID-19 is an unfolding phenomenon, there is currently limited understanding of its total relationship with job insecurity. However, early indications and analyses suggest that workforce anxiety levels have risen globally [20, 22]. COVID-19 has caused structural economic and policy changes that have weakened job security. The current vaccination initiatives in developed nations have not yet widely affected the MENA region, suggesting that organisations will have to maintain their contingency measures in place for the foreseeable future [19, 21]. Hence employees’ anxieties about job security are likely to persist. Some experts warn that workplace and employment security might never return as we knew it before COVID-19. The ‘new normal’ occasioned by the pandemic [22] may lead organisations to create what Teece [23] terms “dynamic capabilities”, enabling organisations to “create, extend, or modify their resource base.”

### Job insecurity

Job insecurity is a perceptual construct that is uncomfortably familiar to employees worldwide as they come to perceive threats to their jobs. It is defined as “perceived powerlessness to maintain desired continuity in a threatened job situation” [1]. In this research, we focus on aspects of job insecurity that are linked to the perceived threat of job loss rather than those pertaining to loss of job features or correlates such as feelings of hopelessness [24, 25]. According to MK Shoss [24], job insecurity can be a consequence of many causes, inclusive of broad economic factors such as organisational restructuring, recession, etc. Further, job insecurity can be the result of job status (such as being employed on a short-term contract) or cognitive vulnerability [24].

Job insecurity represents a considerable threat to individuals, and this is highly stressful. Stress theory is one of the most useful paradigms for understanding the effects of job insecurity, given that employees use personal resources such as energy to counteract the stressful experience of job insecurity [26]. Yet, employees tend to be facing the threat of job loss experience stress intensely because they are ill-equipped to counteract the threat to their job. The ensuing response cycle is distracting and draining, and the ultimate behavioural result is worsened attitudes and subsequent performance deficits. Additionally, job insecurity has been proposed as a breach of the psychological contract between employees and their employers [27]. This implies that employees make efforts in exchange for pay, recognition and other key work-related outcomes. Nevertheless, job insecurity triggers a perception of violation of these expectations and leads to negative attitudes and behaviours in the workplace.

Prior research into broad macro factors such as wartime crisis has shown that war effects disrupt everyday life and economic stability, causing anxieties about job losses [16]. The vast uncertainties stemming from the COVID-19 crisis have increased the prospects of job loss and, thus, should predict increased job insecurity. Therefore, we propose:

#### H1: COVID-19 perceptions among service employees in the MENA region positively predict job insecurity

### Burnout

Consistent with the predictions of stress reactions, we hold that employees will be unable to deploy resources to counteract the COVID-19 impact on job insecurity simply because there are few options to address such a broad macro stressor. Job insecurity will, therefore, be related to adverse effects such as employee burnout. Employee burnout is defined as a psychological syndrome comprised of emotional exhaustion, depersonalisation and reduced personal accomplishment [28]. Burnout has been conceptualised recently as a psychological state [29]. Burnout erodes an employee’s sense of achievement once employees become disconnected from work. Employees become burnt out in the absence of psychological need, satisfaction and rewards [30, 31]. It may be oversimplified to view burnout as mere physical exhaustion owing to workers being over-worked. Instead, burnout occurs when physical exhaustion combines with other psychological circumstances, as suggested above, and the absence of readiness to recognise the actual demand of the work [30, 32]. These represent only a few of the host of consequences that ensue burnout. This vital facet of burnout is well-founded in the employee burnout research [28, 29, 33]. It is broadly recognised that burnout produces considerable adverse implications for both the worker (stress, absence of interest in career development, declining self-assurance, and elevated depression) and the organisation due to diminished productivity, increased labour turnover, and eroded reputation [34, 35].

COVID-19 has already provided evidence of a link between the change caused by the pandemic and rising levels of burnout [36–39]. This derives largely from increased remote working and isolation [36, 40], the burden of work and understaffing [41]. A significant aspect of the existing literature closely related to our study is Bellou and Chatzinikou’s [29], whose conclusion links burnout and organisational change.

As the COVID-19 pandemic continues, burnout reactions are expected, thus providing one of the motivations of the present research. Previous research shows that job insecurity can be one of the primary sources of burnout e.g., [42, 43]. The literature also posits job insecurity as a proxy for external factors to affect other attitudinal and behavioural variables [16]. Further, the most recent studies investigating COVID-19 psychological effects e.g., [44] have not covered the MENA region extensively. Thus, we expect that COVID-19 perceptions effects will be transmitted via job insecurity into burnout. Accordingly, we hypothesise that:

#### H2: job insecurity among service employees in the MENA region positively predicts workplace burnout

### Job satisfaction

The adverse effects of job insecurity on job satisfaction are perhaps one of the most frequently reported findings in the job insecurity literature e.g. [8, 45] including those conducted in the MENA region e.g. [16, 46] and meta-analyses e.g., [2, 5]. COVID-19 has blanketed the globe posing significant challenges to nations, organisations and individuals. Its macro impact on business has precipitated both contractions and delayed business openings, leading to negative human capital implications. The persistent grip of COVID-19 on business output in the MENA region poses a disruptive force perceived by employees as a threat to job insecurity and a stressor about which little can be done. In the previous section, we reasoned how job insecurity perceptions are directly predictive of burnout, and, in this section, we suggest that burnout negatively influences job satisfaction. Employees perceiving their job as insecure are expected to be emotionally more exhausted, less motivated, and far less satisfied with work. Support for this argument is offered by theories dating as far back as Herzberg et al.’s [47] Two-Factor Theory, as well as Deci and Ryan’s [48] Self-Determination Theory, Agnew and White’s [49] General Strain Theory and Robinson and Rousseau’s [50] Psychological Contract Breach theory. Therefore, we anticipate a negative relationship between burnout and job satisfaction. Moreover, recent empirical investigations e.g., [31, 51–53] have reported a significant and negative relationship between job burnout and job satisfaction.

Based on the rationale of the theories and evidence cited above, we further expect that the effect of job insecurity on job satisfaction will be mediated through burnout, thus:

#### H3: burnout among service employees in the MENA region negatively predicts job satisfaction

### Organisational citizenship behaviours

The job insecurity literature has evolved since the 1980s from its initial focus on negative effects on employee attitudes and health to subsequent organisational impacts through behavioural change. While our prior hypotheses show how job insecurity elevates burnout and reduces job satisfaction, in this section, we further reason that job insecurity also negatively influences discretionary employee behaviour in the form of reduced organisational citizenship behaviours (OCB), defined earlier in the paper. Theory and empirical evidence show that job insecurity has negative implications for attitudes and behaviours. Our expectation is supported by meta-analysis [5] that shows job satisfaction as a key antecedent to OCBs as well as a mediator [54] that transmits other variable effects, mainly triggered by job insecurity [55], into OCBs. Employees reduce discretionary contributions because they are stressed, unprepared to address the stress meaningfully, and are conserving resources by not doing things they are not obligated to do, such as helping co-workers, trying their best, and seeking training to build skills. Thus, building on the prior hypotheses, we further anticipate the effect of COVID-19 perceptions will negatively influence OCBs through job satisfaction; thus:

#### H4: job satisfaction among service employees in the MENA region positively predicts OCBs

We further anticipate that COVID-19 perceptions will influence OCBs via a sequence of mediators composed of job insecurity to burnout to job satisfaction:

#### H5: COVID-19 perceptions among service employees in the MENA region have indirect adverse effects on OCBs via a series of mediators following the order: job insecurity, burnout and job satisfaction

### Differences among generational cohorts

In this study, we reference several generational cohorts. Baby Boomers (born between 1946 and 1964); Generation X (born between 1965 and 1979); Generation Y born between (1980 and 1994), and Generation Z (born after 1995) [56, 57]. Generational cohorts have previously been examined in a workplace context [58]. A generational cohort includes an identifiable group of individuals who share distinctive social or historical life events during critical developmental stages [58]. However, the perspective about close similarities in generations has been questioned in recent research [59]. CW Rudolph and H Zacher [59], for instance, suggest that work processes and outcomes are not substantially affected by generational variations. Current developments may give some credence to this argument. In fact, economic development and globalisation make today’s workplace more complex than ever before, and improved general health conditions lead to prolonged career life for employees [60], thus providing a rich context for this study. We, therefore, cautiously use the generational groupings for the purpose of conceptual consistency with mainstream sociological literature while acknowledging the theoretical limitation highlighted by CW Rudolph and H Zacher [59].

Baby boomers are providing an excellent opportunity for younger generations to play a more significant role in the workplace upon the boomers’ retirement at a record rate and pace [61]. However, as per Rudolph and Zacher’s [59] finding earlier cited, generational differences do not necessarily impact work outcomes. Their main effects lie in work values, expectations and attitudes, which could generate potential conflict and affect readiness for change [57]. In fact, unlike their Baby boomer managers and supervisors, who were prone to working long hours, Generation X employees tend to value work-life balance, making sure they have more time to dedicate to their families. Generation X is characterised as self-directed, sceptical and autonomous, born during a time of rapid change. While they are looking for work-life balance, they are not impressed by authority and micromanagement [62]. In contrast, Generations Y and Z - 61 to 77% in a large survey [57] – would engage in more aggressive paths to satisfy their leadership ambitions, often taking more significant risks, compared with only 57% of Generation X cohorts.

As their ancestors (i.e., the Baby Boomers) become retirees and leave the workplace clear for their descendants, Generation X employees are becoming senior staff members in the workplace, with their offspring (Generation Z) climbing the ranks [63]. As a result, many individuals of Generation X have interests in social media and cell phones that are similar to those of younger generations. However, generation X employees tend to have different patterns of communication inclinations from those of younger generational cohorts. For instance, Generation Z employees have a preference for using texting instead of e-mail in order to communicate with colleagues in the workplace— a method not ideal for Generation X [63].

According to The Federal Competitiveness and Statistics Authority (FCSA), Generation Y is turning into the workforce’s largest cohort in the MENA region. For example, 58 and 64% of the UAE and Oman workforce were Millennials [64, 65]. In addition, both Generation Y and Generation Z employees are tech-native [66, 67]. Further, Generation Y is often perceived as connected, self-confident and agile [68].

Generation Z is now the youngest generation joining the manpower. Their utilisation of technology and quest for flexibility in the employment arrangements are largely similar to the Millennials [69]. Generation Z employees recognise the significance of financial security and are known for their thrill of excelling at work and desire for professional accomplishment [67, 70]. Moreover, both Generation Y and Generation Z have been shown to be more ethnically diverse as compared to all of the previous generational groups [57, 61, 70, 71].

Our study contends that realising the generational divide in the workplace is crucial because it can lead to conflict and poor levels of engagement amongst both employees and management. Conversely, when generational disparities are effectively addressed, a healthy workplace will be fostered, and employee motivation and engagement strengthened. Our study’s hypothesised model introduces an attempt to analyse the potential pandemic perceptions effects on OCBs via a series of attitudinal variables, namely job insecurity, burnout, and job satisfaction. OCBs are valuable extra-role behaviours that support organisational goals. Nevertheless, in a formal sense, employees are not required by employers to engage in OCBs, e.g. helping colleagues, sharing knowledge, talking favourably about the organisation to outsiders, etc. [72]. Therefore, we seek to understand the indirect relationship between COVID-19 perceptions and OCBs via a sequence of transmitters or mediating variables (job insecurity to burnout to job satisfaction) while adding multigenerational complexities which may impact the relationships studied here [73]. This study will contribute to our scholarly understanding of the need to tackle generations differently in organisational response to pandemic-driven job insecurity.

Based on the previous studies related to cognitive, attitudinal and behavioural variances across generational cohorts in the workplace e.g. [67, 74–77], we have witnessed rising concerns about the generational variances of emotional fragility when triggered by the perception of job stressors e.g., [78, 79] especially during a pandemic time [80–82]. Furthermore, a recent large survey [57] of over 19,000 people across several countries found striking differences between generations, which led the authors to suggest that organisations are compelled to keep those differences in mind. Such differences include variations in aspirations, values, needs and expectations. For example, with regards to the use of modern work-related technologies such as virtual reality (VR), which could significantly enhance one’s ability to reskill or upskill themselves, Generation X showed significant reluctance compared with the enthusiasm shown by Generation Z and, to some extent, by Generation Y [57]. Also, we respond to the call [67] for investigating generational differences concerning the relationship between COVID-triggered job insecurity and OCB in non-Western contexts. Thus, we expect generational differences will result in inconsistencies in employees’ COVID-19 perception effects on OCBs transmitted via job insecurity, burnout, and job satisfaction. We hypothesise:

#### H6: generation moderates the relationships between COVID-19 perceptions and OCBs among service employees in the MENA region

**Fig. 1:**
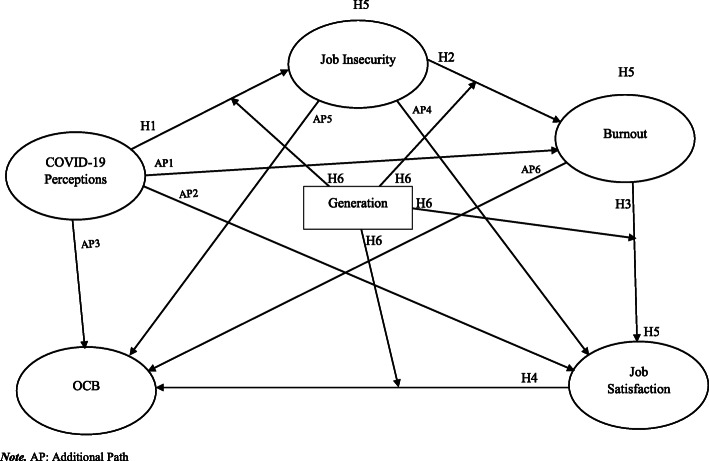
Hypothetical model

To determine whether parallel mediations could exist, we hypothesise additional direct paths: COVID-19 Perceptions to Burnout, Covid-19 Perceptions to Job Satisfaction, COVID-19 Perceptions to OCB, Job Insecurity to Job Satisfaction, Job Insecurity to OCB, and Burnout to OCB. If they were found significant and sizable, the mediations, if statistically supported, would be deemed partial, otherwise full [77, 83–85]. The research model is presented in Fig. 1.

## Method

### Participants

The MENA region occupies a strategically important geographic location between Europe, Africa and Asia. The population currently exceeds 450 million, or 6% of the world population [10]. Our study population consisted of customer service employees in the MENA region, where we randomly selected three countries, i.e., the United Arab Emirates, Egypt and Oman, out of the region’s twenty countries. While customer service employees’ perceptions and behavioural attitudes in the workplace have been shown to be instrumental for service industries’ jobs; they play a central and integral part in the service delivery [86], where employee interactions with the customer (internal or external) are directly linked to long-term economic sustainability.

### Research design

Our research is quantitative. It adopts a cross-sectional survey design study that supports post-empiricism assumptions and draws on social constructionism [87] to form an understanding of our sample’s attitudinal and behavioural tendencies.

### Analysis

We adopted several indicators to evaluate the validity and reliability of measures employed in this study [85]. First, we tested the hypothetical model utilising a PLS-SEM approach method via SmartPLS 3 [88]. Prior research [89, 90] has recommended employing the PLS-SEM method for evaluating predictive models, which is why we chose this strategy. Furthermore, according to previous research [91], multivariate normality is likely to be violated by most data. Besides, PLS-SEM has also gained widespread acceptance in empirical investigations when data are vulnerable to non-normality bewilderment [92], as shown by an ever-growing amount of literature endorsing it. Furthermore, the PLS-SEM is becoming increasingly widely used and recognised in the fields of work and health psychology [17] and public health [93]. Second, we conducted a multi-group analysis (MGA), building on standardised betas (β: for direct effects), un-standardised betas (B: for indirect effects) and the matching t-values by using bootstrapping, Q^2^ for predictive relevance and Cohen’s f^2^ to ascertain effect sizes where f^2^ ≥ .02, f^2^ ≥ .15, and f^2^ ≥ .35 “indicate small, medium and large effect sizes, respectively” [94]. Besides, standard root mean square residual (SRMR) was used to evaluate the model fit to the data [95].

### Measurement of variables

Appendix 1 shows a set of already validated measures that we utilised in this study. We used previously validated scales reported in the work of AB Mahmoud, D Hack-Polay, L Fuxman and M Nicoletti [17] to measure COVID-19 perceptions, L Francis and J Barling [96] to measure job insecurity, D Lang [97] and C Maslach and SE Jackson [28] to measure burnout, TA Judge, BA Scott and R Ilies [98] to measure job satisfaction, and finally, L Van Dyne, JW Graham and RM Dienesch [99] to measure organisational citizenship behaviour. All measures were evaluated on a 5-point Likert scale. Also, all of the measures, including the COVID-19 perceptions, fulfilled the reliability and discriminant, convergent and construct validity criteria as exhibited in the results section. We calculated the Heterotrait-Monotrait Ratio of Correlations (HTMT), and they are presented in Table 1. We found that all of the correlations had values less than .9 implying that all of the measures had a satisfactory discriminant validity [100]. Table 2 indicates that all the constructs had average variance extracted scores (AVEs) higher than .5 [101], composite reliability values (CRs) between .7 and .9 [85] satisfying the convergent validity and reliability criteria for all of the measures [100]. Moreover, Table 2 shows that the Variance Inflation Factor values for all the measures’ items were less than 5 offering evidence that collinearity is not a crucial issue [100].

**Table 1:** Discriminant validity test (HTMT)

	Burnout	Covid-19 Perceptions	Job Insecurity	Job Satisfaction
**Covid-19 Perceptions**	0.264			
**Job Insecurity**	0.521	0.397		
**Job Satisfaction**	0.449	0.164	0.269	
**OCB**	0.109	0.142	0.13	0.586

**Table 2:** Outer loadings, VIFs, construct reliability and validity and descriptive statistics

	Burnout	Covid-19 Perceptions	Job Insecurity	Job Satisfaction	OCB	VIF
**JSEC01**			0.825			1.538
**JSEC02**			0.717			1.819
**JSEC03**			0.671			1.932
**JSEC04**			0.674			1.627
**BURNOUT01**	0.754					1.547
**BURNOUT02**	0.704					1.926
**BURNOUT03**	0.743					1.585
**COV01**		0.815				3.651
**COV02**		0.741				1.300
**COV03**		0.711				3.306
**JS01**				0.728		1.523
**JS02**				0.821		1.923
**JS03**				0.605		2.541
**JS04**				0.868		2.299
**OCB01**					0.745	1.756
**OCB02**					0.643	2.185
**OCB03**					0.860	2.038
**OCB04**					0.764	1.844
**OCB05**					0.566	1.713
**Cronbach’s Alpha**	0.779	0.797	0.817	0.841	0.846	
**rho_A**	0.779	0.804	0.821	0.859	0.856	
**Composite Reliability**	0.778	0.8	0.814	0.845	0.843	
**AVE**	0.539	0.573	0.525	0.581	0.522	
**Mean**	3.030	3.478	3.086	3.313	4.120	
**SD**	1.190	1.102	1.163	1.019	0.880	

### Sampling techniques and sample description

The data analysed in this study were part of an ongoing participants recruitment process in three countries in the MENA region consisting of Egypt, Oman and the United Arab Emirates. That part represented data collected from the beginning of April to the end of June 2020. The primary approach to finding participants was through LinkedIn (a professional social network). We hired professional surveyors who set the search filter criteria to identify participants from the target population. Aiming for a sample size that would contain considerable numbers of each generational cohort, we approached more survey participants through other online social platforms and networks like WhatsApp, Facebook, etc. [89]. The survey did not include a question identifying the participants’ country of residence. All the participants were made aware and cognizant of the aim, objectives and procedures of the investigation. Participants were informed that they could ask questions, express issues about the survey, and leave the study at any stage of the survey. The questionnaire also included an agreement to participate in the survey. Because the study was performed online, the participants’ signatures were not obtained. The survey responses were anonymously recorded, and all participants were informed that their answers would be treated with confidentiality. It took about ten minutes to complete the questionnaire, which was made available for the participants in Arabic and English. Given that all measures used in this study were initially constructed in an English-speaking Western setting, all measures and scales underwent double translation (i.e., English to Arabic and then Arabic to English). This is a well-established method for removing meanings that are not intended in a foreign version of a measure [46]. After performing this procedure, further efforts were made to establish the face validity for the measures. The scales were shared with Arabic-speaking academics and practitioners in human resource management to assess the Arabic phrasing in the questionnaire items and suggest corrections where needed. Besides, the questionnaire was piloted to a convenience sample of thirty Arabic-speaking employees in Egypt to assure the understandability of the measures’ items. Overall, our study yielded 578 responses. We computed the generation variable by recoding participants’ age using the generational thresholds identified in previous research.

The final sample was composed of three generational groups, i.e., Generation X (32%), Generation Y (45%) and Generation Z (23%). Fifty-eight per cent of our sample were male. Further, many of our participants were educated to a university degree (41%) and single (55%). Appendix 1 shows the descriptive statistics of the variables under investigation clustered into generational groups. Appendix 2 demonstrates the descriptive statistics (produced using SPSS version 26) of the path model constructs for the whole sample and each generational group.

## Results

### Common method bias

Before moving on to the path and multi-group analyses, we ran Common-Method Bias (CMB) tests, which are required when using perceptual, self-report measures from a single survey [102]. The inner variance inflation factors (VIFs) values (see Table 3) were all less than 3.3 [103]. Hence, there were no CMB issues identified.

**Table 3:** Inner VIFs values

	Burnout	Covid-19 Perceptions	Job Insecurity	Job Satisfaction	OCB
**Burnout**		1.427			
**Job Insecurity**		1.207			
**Job Satisfaction**		1.617			
**OCB**		1.396			
**Burnout**			1.228		
**Covid-19 Perceptions**			1.047		
**Job Satisfaction**			1.321		
**OCB**			1.141		
**Burnout**					
**Covid-19 Perceptions**	1.128				
**Job Insecurity**	1.198				
**Job Satisfaction**	1.374				
**OCB**	1.313				
**Burnout**				1.202	
**Covid-19 Perceptions**				1.131	
**Job Insecurity**				1.239	
**OCB**				1.030	
**Burnout**					1.126
**Covid-19 Perceptions**					1.114
**Job Insecurity**					1.133
**Job Satisfaction**					1.073

### Path analysis

We performed Consistent-PLS Algorithm, followed by Consistent PLS Bootstrapping run at 5000 sub-samples [104]. As a result, COVID-19 perceptions are discovered to positively predict job insecurity (β = .400, *P* < .001, f^2^ > .15) that in turn positively predicts burnout (β = .526, *P* < .001, f^2^ > .35). Burnout is found to negatively relate to job satisfaction (β = −.440, *P* < .001, f^2^ > .15). Ultimately, job satisfaction is found to positively predict OCB (β = .595, *P* < .001, f^2^ > .35). Therefore, we judge that H1, H2, H3 and H4 are fully supported (See Table 4). Moreover, Table 4 demonstrates that none of the additional paths, i.e., COVID-19 Perceptions to Burnout (β = .086, *P* = .147, f^2^ < .02), Covid-19 Perceptions to Job Satisfaction (β = −.063, *P* = .340, f^2^ < .02), Covid-19 Perceptions to OCB (β = −.064, *P* = .334, f^2^ < .02), Job Insecurity to Job Satisfaction (β = .008, *P* =. 878, f^2^ < .02), Job Insecurity to OCB (β = .065, *P* = .161, f^2^ < .02), and Burnout to OCB (β = −.013, *P* = .811, f^2^ < .02), are found significant and sizable, therefore, indirect effects are to be judged as full mediations if they are found significant.

**Table 4:** Hypotheses 1–4 testing – direct effects

Hypothesis	Path	β	***t***	f^**2**^	Decision
H1	Covid-19 Perceptions - > Job Insecurity	0.400^**^	9.716^**^	> 0.15	Supported
H2	Job Insecurity - > Burnout	0.526^**^	13.789^**^	> 0.35	Supported
H3	Burnout - > Job Satisfaction	−0.440^**^	10.503^**^	> 0.15	Supported
H4	Job Satisfaction - > OCB	0.595^**^	20.523^**^	> 0.35	Supported
Additional path1	Covid-19 Perceptions - > Burnout	0.086^*NS*^	1.443 ^*NS*^	< 0.02	Unsupported
Additional path2	Covid-19 Perceptions - > Job Satisfaction	−0.063 ^*NS*^	0.95 ^*NS*^	< 0.02	Unsupported
Additional path3	Covid-19 Perceptions - > OCB	−0.064 ^*NS*^	1.321 ^*NS*^	< 0.02	Unsupported
Additional path4	Job Insecurity - > Job Satisfaction	0.008 ^*NS*^	0.152 ^*NS*^	< 0.02	Unsupported
Additional path5	Job Insecurity - > OCB	0.065 ^*NS*^	1.423 ^*NS*^	< 0.02	Unsupported
Additional path6	Burnout - > OCB	−0.013 ^*NS*^	0.24 ^*NS*^	< 0.02	Unsupported

** *P* < .001; *NS*: Non-significant

Table 5 shows that all the un-standardised betas are significant at a probability value less than .001; thus, we conclude that H5 is fully supported. This finding suggests that, although COVID-19 perceptions do not directly affect OCB, their damaging effects indirectly navigate towards OCB. These adverse effects of COVID-19 perceptions are *fully* transmitted via a sequence of mediators, i.e., job insecurity to burnout to job satisfaction. Put it in other terms, more intense COVID-19 perceptions will increase job insecurity which will subsequently level up burnout (B = .211, SD = .028, *P* < .001) that will, in turn, adversely affect job satisfaction (B = −.093, SD = .015, *P* < .001) and therefore lead to fewer chances of employees engaging in OCB (B = −.055, SD = .009, *P* < .001). Finally, with SRMR equivalent to .043 < .08, we judge our hypothetical model as an excellent fit for our data [105]. Table 6 shows that the Q^2^ values of all the predictors are greater than 0, which implies adequate predictive relevance. Also, R^2^ values for job insecurity (.16), burnout (.28), job satisfaction (.19) and OCB (.36) were all above zero, suggesting that our model has substantial predictive accuracy [94].

**Table 5:** Hypothesis 5 testing – indirect effects

Path	B	STDEV	t
Covid-19 Perceptions - > Job Insecurity - > Burnout	0.211^**^	0.028	7.536^**^
Job Insecurity - > Burnout - > Job Satisfaction	− 0.231^**^	0.028	8.201^**^
Covid-19 Perceptions - > Job Insecurity - > Burnout - > Job Satisfaction	− 0.093^**^	0.015	6.144^**^
Burnout - > Job Satisfaction - > OCB	−0.261^**^	0.026	10.090^**^
Job Insecurity - > Burnout - > Job Satisfaction - > OCB	−0.138^**^	0.017	7.880^**^
Covid-19 Perceptions - > Job Insecurity - > Burnout - > Job Satisfaction - > OCB	− 0.055^**^	0.009	5.939^**^

** *P* < .001

**Table 6:** Predictive relevance (Q^2^)

	SSO	SSE	Q^**2**^ (=1-SSE/SSO)
**Burnout**	2541.00	2252.55	0.114
**Job Insecurity**	3388.00	3173.23	0.063
**Job Satisfaction**	3388.00	3105.71	0.083
**OCB**	4235.00	3594.57	0.151

### Measurement invariance of the composite models

Before examining the moderating role of generation, we ran Measurement Invariance of the Composite Models (MICOM), as suggested by J Henseler, R-JBJ R. Sinkovics, R Daekwan Kim, CM Ringle and M Sarstedt [106], to test whether both configural invariance and compositional invariance were established. As indicated earlier, we employed a variance-based approach; thus, according to [107], configural invariance was verified by default. Moreover, our permutation test (see Table 7) returned *p*-values greater than .05. This led us to accept the null hypothesis (the constructs’ original correlations are non-significantly different from 1), offering evidence of compositional equivalency and supporting the viability of conducting multi-group analysis [107].

**Table 7:** Compositional invariance assessment

	Original Correlation	Correlation Permutation Mean	5%	Permutation p-Values
**X vs Y**				
Burnout	1.000	0.993	0.979	0.948
Covid-19 Perceptions	1.000	1.000	1.000	0.240
Job Insecurity	0.999	0.994	0.983	0.784
Job Satisfaction	1.000	1.000	0.999	0.850
OCB	0.995	0.984	0.957	0.700
**X vs Z**				
Burnout	0.992	0.953	0.926	0.640
Covid-19 Perceptions	1.000	1.000	1.000	0.370
Job Insecurity	0.998	0.930	0.932	0.852
Job Satisfaction	0.999	0.999	0.997	0.400
OCB	0.943	0.965	0.891	0.122
**Y vs Z**				
Burnout	0.995	0.985	0.964	0.386
Covid-19 Perceptions	0.948	0.927	0.799	0.150
Job Insecurity	0.995	0.953	0.965	0.390
Job Satisfaction	1.000	0.999	0.998	0.436
OCB	0.928	0.930	0.680	0.186

### Multigroup analysis

**Fig. 2:**
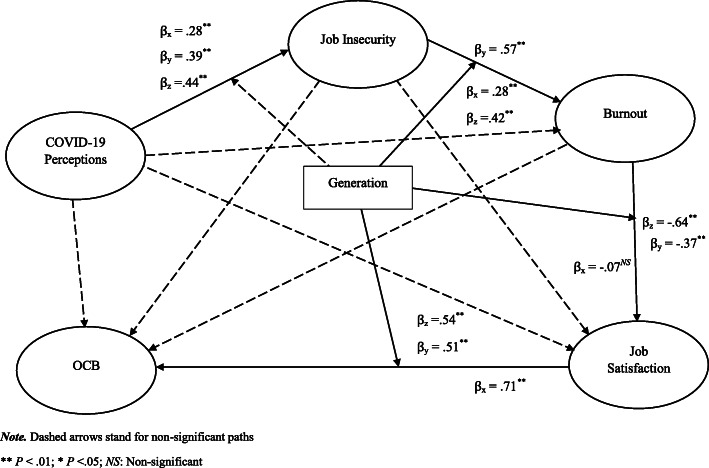
Path model analysis

To assess the path model invariance across generational cohorts, we ran a multi-group analysis (MGA). Using the t-values associated with the multiple comparisons and reported in the parametric tests, we found the paths: job insecurity to burnout, burnout to job satisfaction, and job satisfaction to OCB were substantially non-equivalent across Generations X, Y, and Z. Figure 2 illustrates the paths moderated by generational differences. More detailed, Table 8 shows that generation Y (β_y_ = 0.57; *P* < .01) levels of burnout tend to be more susceptible to variations in perceived job insecurity than any other generational group in our sample. Only younger generations, Generation Z (β_z_ = − 0.638; *P* < .01) and Generation Y (β_y_ = − 0.374; *P* < .01) tend to experience slumps in job satisfaction as the levels of burnout are amplified. This means that Generation X (β_x_ = − 0.066; *P* = .638) is considerably less likely to experience a lack of job satisfaction than younger generations because of burnout. Job satisfaction amongst Generation X (see Table 9) seems to block the cascading effects of COVID-19 perceptions on OCB (B = − 0.003; *P* = .621), which is not the case for younger generations where COVID-19 perceptions damages extend to job satisfaction (B_y_ = −.083; *P* < .01; B_z_ = −.012; *P* < .01) and OCB (B_y_ = −.042; *P* < .01; B_z_ = −.064; *P* < .01). This implies that younger generations are substantially more vulnerable to the indirect effects of COVID-19 perceptions than Generation X. With the relationship between COVID-19 and job insecurity not moderated by generation whilst the remaining three paths are either directly or indirectly moderated by generation, we arrive at the conclusion that H6 is partially supported.

**Table 8:** Hypothesis 6 testing – Multigroup invariance analysis of direct effects

Path	β_**X**_	β_**Y**_	β_**Z**_	t-Value(Gen X vs Gen Y)	t-Value(Gen X vs Gen Z)	t-Value(Gen Y vs Gen Z)
Covid-19 Perceptions - > Job Insecurity	0.276^**^	0.389^**^	0.436^**^	1.618 ^*NS*^	1.933 ^*NS*^	0.476 ^*NS*^
Job Insecurity - > Burnout	0.277^**^	0.572^**^	0.424^**^	4.041^**^	1.369^*NS*^	2.308^*^
Burnout - > Job Satisfaction	−0.066 ^*NS*^	−0.374^**^	−0.638^**^	2.51^*^	3.581^**^	3.381^**^
Job Satisfaction - > OCB	0.709^**^	0.511^**^	0.543^**^	3.734^**^	3.717^**^	0.434

* *P* < .05; ** *P* < .01; *NS* = Non-significant

**Table 9:** Hypothesis 6 testing – Multigroup invariance analysis of specific indirect effects

Path	B_**X**_	B_**Y**_	B_**Z**_	STDEV (Gen X)	STDEV (Gen Y)	STDEV (Gen Z)	***t***-Value(Gen X vs Gen Y)	***t***-Value(Gen X vs Gen Z)	***t***-Value(Gen Y vs Gen Z)
Covid-19 Perceptions - > Burnout	0.076^**^	0.222^**^	0.185^**^	0.025	0.03	0.036	3.517^**^	2.424^*^	0.864 ^*NS*^
Covid-19 Perceptions - > Job Satisfaction	−0.004^*NS*^	−0.083^**^	− 0.118^**^	0.009	0.017	0.027	3.618^**^	4.352^**^	1.01 ^*NS*^
Covid-19 Perceptions - > OCB	−0.003^*NS*^	−0.042^**^	− 0.064^**^	0.006	0.009	0.016	3.159^**^	3.714^**^	1.092 ^*NS*^
Job Insecurity - > Job Satisfaction	−0.014^*NS*^	−0.214^**^	− 0.271^**^	0.032	0.033	0.05	4.052^**^	4.422^**^	0.907 ^*NS*^
Job Insecurity - > OCB	−0.009^*NS*^	−0.109^**^	− 0.147^**^	0.023	0.019	0.029	3.248^**^	3.569^**^	1.02 ^*NS*^
Burnout - > OCB	−0.046^*NS*^	−0.191^**^	− 0.346^**^	0.091	0.029	0.033	1.748 ^*NS*^	2.617^**^	3.219^**^

* *P* < .05; ** *P* < .01; *NS* = Non-significant

## Discussion

Our study empirically examined a path model between COVID-19 perceptions and OCBs via job insecurity, burnout and job satisfaction as mediators. Our findings revealed that a sequence of three mediators: job insecurity, burnout and job satisfaction, mediates the relationship between COVID-19 perceptions and OCBs. As expected, we found a positive correlation between COVID-19 perceptions and job insecurity, job insecurity and burnout, a negative correlation between burnout and job satisfaction. Job satisfaction was found to be positively correlated to OCBs (supporting hypotheses 1–5). Furthermore, our model confirmed that COVID-19 perceptions exerted a negative indirect effect on OCB. This means the perceptions of COVID-19 will elevate burnout by triggering job insecurity. Increases in the levels of workplace burnout will decrease OCBs via eroded job satisfaction amongst employees.

Our findings expand upon prior research that examined broad macro influences like wartime conditions on job insecurity. We then further investigated the moderating impact of generational differences on the whole path from COVID-19 perceptions to OCBs amongst customer service employees in three countries in the MENA region. Our generational analysis reveals the existence of some significant cross-generational variations. Generation X employees are shown to be more likely to increase their involvement in OCBs when they are satisfied with their job; this is not as discernible as it seems amongst younger generations— both Generation Y and Generation Z workers. Our results suggest that this can be a result of the hindrance role of job satisfaction which blocks the cascading effects of burnout amongst Generation X. The intensity of the connection between increased levels of COVID-19 perceptions and lower engagement in OCBs is amplified amongst employees of younger generations compared to those of the older Generation X. When generational variations are understood and successfully managed, particularly for customer service employees, increased employee engagement and improved motivation will lead to a more balanced and healthier workplace, which in turn translate into customer satisfaction and loyalty as suggested by the service profit chain model [77].

### Research implications

The workplace in contemporary organisations requires researchers to study differences attributable to demographic factors such as generational composition. In addition, firms seek practical guidance that will help stimulate optimal performance and sustain valuable discretionary behaviours such as OCBs. Therefore, we need research that identifies patterns of consistency or points to divergent patterns among different generations.

The current study found that COVID-19 perceptions can be classified as a crisis, much as that of wartime-like crisis, and this causes indirect harm to employees’ willingness to perform non-formal requirements of the work [16, 108]. Interestingly, we found that COVID-19 perceptions had a similar positive association with job insecurity across the three generations. This result further supports the idea that no one is safe concerning the perceived threat to their job security when triggered by COVID-19; even the greater propensity of Generations Y and Z to contemplate paths to leadership and willingness to take risks does not shield them from threat to job security. However, the subsequent attitudinal and behavioural consequences of job insecurity were found to vary across generations. Our results suggest that younger generations, especially the Millennials, are more vulnerable to developing burnout as a result of pandemic-caused job insecurity than other generations. This vulnerability may be amplified in the MENA cultural context where greater credence is accorded to age and seniority, meaning potentially more protection or job security for Generation X cohorts [109] and high unemployment rates for younger people. This result is consistent with the current discourse on employment, where Millennials are being used metaphorically to depict employees’ vulnerability to the growing precarity of work amidst the COVID-19 pandemic. For instance, J Filipovic [110] subtitles her article ‘we’re all Millennials now’ to describe the deteriorating work conditions like job insecurity, low pay and working from home alongside the adverse emotional ramifications that have been introduced by modern technology and virtual workplaces. Such pandemic-time consequences have not been limited to the Millennials but have also affected the workforce from all generations [110].

Another important finding is that, unlike Generations Y and Z, Generation Xers’ COVID-19 perceptions’ indirect effects onto OCBs were found non-significant. This finding accords with our analysis which revealed that job satisfaction was not significantly related to burnout amongst Generation X employees suggesting that job satisfaction buffers the trickling effects of COVID-19 perceptions onto OCBs through the sequence of mediators— job insecurity, burnout and satisfaction. Moreover, this further explains why satisfied Generation Xers are substantially more likely to perform OCBs than younger generations. Besides, these results are in keeping with previous observational surveys about mental health across generations. For example, a recent report [111] revealed that as Millennials age, the generation is witnessing much faster declines in mental and physical health than those from Generation X. Besides, the report warned that unless proper management or treatment are developed and implemented, the Millennials could see a 40% surge in mortality rate in comparison with the Generation Xers of the same age [111].

In view of the variations amongst generational groups, developing workplace communication methods to minimise conflict between employees is becoming increasingly vital through employing motivation-based communication strategies. Therefore, managers need to communicate with workers in ways that would define *work* as a “positive experience” [77, 89]. That would help protect employees’ job satisfaction and, consequently, their levels of engagement in OCBs as an outcome of controlled burnout, as per our findings. This means that multi-generation organisations need to pay attention to the implications of the disparities in the tools used for communication. For instance, when Generation X employees favour e-mailing over other tools for communication, they might frown upon Generation Z employees’ taste for texting [63] which can elevate the chances of organisational conflicts, particularly as the traditional workplace environment has been replaced by largely virtual ones almost overnight during the pandemic.

We examined the impact of employee’s COVID-19 perceptions on the extent of employee’s engagement in OCBs and found that it is non-equivalent to the generational differences. This study contributes to our understanding of how times of pandemic can affect OCBs as we tested a path model linking COVID-19 perceptions to OCBs via employee’s job insecurity, burnout and job satisfaction as mediating factors. Another key research finding is acknowledging generational issues in the workforce, which can engender major fluctuations in behaviour in terms of the responses to increased COVID-19 perception from different generation groups.

The study showed that workers among the Generation Y and Generation Z cohorts unsurprisingly have a greater propensity to withdraw from OCBs if they have apprehension about job insecurity, which causes them to develop burnout symptoms at a faster pace compared to Generation X cohorts. Generally, the individual and organisational consequences of higher job insecurity and burnout tend to be less of a concern for workers within Generation X. An explanation for this may be linked to the buffering role of job satisfaction amongst Generation X workers as well as the fact that younger generational cohorts can be more vulnerable to job stressors and hence be at higher risk to disengage in OCB. Besides, younger generations show more flexibility and willingness to engage with changing job contexts and relocating, aiming for more secure and stable jobs e.g., [110]. Most chief financial officers in the MENA region (e.g., 39% in the UAE) continue to see adverse changes in staffing and layoffs [112]. Unemployment in Egypt has risen to 9.6% in the second quarter of 2020, compared to 7.5% in the same period the previous year, and it is thought that the COVID-19 crisis may amplify poverty rates in the country to 40% from 30% [113]. Thus, the extent of job insecurity could continue to rise during the period of the pandemic. Based on our findings, it is anticipated that this would have negative ramifications for individuals, societies, and governments.

A significant endeavour for organisations is to strive to retain the experience of their Generation X employees. These have shown loyalty and have a wealth of skills [114] that could be deployed in various ways. For example, they could act as potent external consultants due to their inside knowledge of the organisation. What is needed is to reassure Generation Xers about their continuous importance to organisational continuity. This way, organisations will strike a balance between nurturing Millennials and rewarding Generation Xers, who will continue to ensure the retention of organisational knowledge [114]. In the context of COVID-19, human resource management (HRM) has an important role to play in providing counselling, advice and guidance to all employees to ensure that they do not disengage. HRM also has a responsibility to design work and contract patterns that create room for flexibility. For example, the recent furlough strategy (reduction in hours or pay and temporary layoffs) adopted by many companies during the COVID-19 pandemic was geared to keeping the employees connected and ready for re-hiring [115].

### Research limitations and implications

This study employed the traditional classification of generations that has been broadly adopted in current social psychology and public health scholarship, including those conducted in the MENA region e.g., [89]. While this research has successfully shown that generational differences lead to variations in the hypothesised relationships, it has, nevertheless, a particular limitation about the probability that such differences may also be generated by the age or status of the corresponding generational cohort. Recent scholarly work [116, 117] has called for abandoning the concept of generations and generational differences and instead exploring alternative lifespan development conceptual frameworks that are more accurate. Therefore, we suggest that further research should address this matter. Recent research on generational differences in the workplace [67] encourages looking at whether or not Generation Z employees’ attitudinal and behavioural patterns might turn out within the next two decades to be more analogous to Generation Y’s in the 2010s. Alternatively, whether Generation Alpha (entering the job market in 2030) would demonstrate the same characteristics as Generation Z today. If yes, that would suggest that such variations in organisational attitudes and behaviours were age-caused rather than generational. If not, that would earn the generational cohort’s impact more legitimacy in triggering attitudinal and behavioural differences in the workplace.

A further limitation of the study is its sampling. The convenience sampling used is principally motivated by the current pandemic situation and therefore is not fully representative. This study sample description was also limited by the absence of a question identifying the respondents’ current country of residence. Although the selection of three countries was aimed primarily for better representation for the MENA region, however, including that question would have allowed for richer profiling for our sample. Further, the limitation of the study with regards to data collection solely from the MENA region warrants a re-examination of the effects of COVID-19 perceptions on job attitudes and organisational outcomes and the moderating role of generation in additional geographical settings. It is also recommended that future researchers examine multi-cultural, multi-gender, and multi-industry refinements to the model. That will further enrich our understanding of employees’ contextual performance and discretionary behaviours for different genders and across several different employment sectors. Moreover, the countries’ economic development, levels and speed of technology adoption and advancement embedded in service jobs might all moderate the complex dynamics of the impact of pandemic-triggered job insecurities on OCBs among a diverse workforce. Future studies may be able to integrate some or all these factors to explore potential moderations in our model.

One thing that might produce response bias in the answers obtained and analysed in this study is the employment of self-reporting data [118]. We acknowledge this limitation because this type of data, tacitly, suggests that the respondents have the same comprehension or interpretation of the survey items [55]. The research is also limited by the cross-sectional design, so causal effects are implied but not formally evaluated. Therefore, future research is encouraged to collect longitudinal data to understand the real effects and causal relationships better. However, employing a longitudinal design to evidence causality has been amplified [119]. It only offers limited advantages over the cross-sectional design in most cases in which it is used [119]. Additionally, consistent with P Tharenou, R Donohue and B Cooper [120], discoveries made out of cross-sectional research can still be interpretable and legitimate if a robust theoretical basis is embraced. Moreover, cross-sectional design for data collection has received backing for scholarly inquiries in wartime-like circumstances (e.g., COVID-19) and other contexts of extreme environments e.g. [121].

## Conclusion

This essay has discussed the COVID-19 perceptions pronounced effects on OCB travelling through increases in job insecurity and burnout, ultimately worsening job satisfaction. More importantly, this paper has argued that those effects, landing indirectly into OCB, are non-equivalent across generational cohorts in the MENA region. With regards to the indirect impacts of COVID-19 perceptions on their participation in OCBs, younger generations are much more susceptible than older generations. On the other hand, Generation X work satisfaction mitigates the effects of COVID-19 perceptions on OCBs. Therefore, the current research contributes to our understanding of generational disparities in the workplace when it comes to reacting to perceived health crises or pandemics.

## Supplementary Information

**Additional file 1.**

## Data Availability

The data used in this study will not be available due to confidentiality issues. Any queries regarding data availability should be directed to Dr. Ali B Mahmoud: elguitarrista@live.com.
