# Supplementary material for: Who’smore vulnerable? A generational investigation of COVID-19 perceptions’ effect on Organisational citizenship Behaviours in the MENA region: job insecurity, burnout and job satisfaction as mediators
Source: BMC Public Health. 2021 Oct 27;21:1951. doi: 10.1186/s12889-021-11976-2 (PMC8549417; doi:10.1186/s12889-021-11976-2)
Supplement: Supplementary file 1 — Additional file 1. [file 12889_2021_11976_MOESM1_ESM.docx]

**Supplementary materials**

**Appendix 1: Measures used in the current study**

| **Variable** | **Item** | **Assessment** | **Source** |
| --- | --- | --- | --- |
| **Burnout** | I feel emotionally drained from my work | 5-point Likert scale. 1 = "strongly disagree," 5 = "strongly agree" | C Maslach and SE Jackson [28] |
|  | Working with people directly puts too much stress on me |  |  |
|  | I’ve become more callous toward people since I took this job |  |  |
| **Job Satisfaction** | I am enthusiastic about my work | 5-point Likert scale. 1 = "strongly disagree," 5 = "strongly agree" | TA Judge, BA Scott and R Ilies [98] |
|  | At this moment, I am finding real enjoyment in my work |  |  |
|  | Overall, I like my job |  |  |
|  | Right now, I consider my job rather unpleasant* |  |  |
| **Job Insecurity** | The possibility of losing my job occupies my thoughts constantly. | 5-point Likert scale. 1 = "strongly disagree," 5 = "strongly agree" | L Francis and J Barling [96] |
|  | No matter how hard I work there is no guarantee that I am going to keep my job. |  |  |
|  | I am certain of losing my job. |  |  |
|  | The probability of being laid-off is high. |  |  |
| **Covid-19 Perceptions** | I believe that the effect the coronavirus pandemic has had on people is | 5-point Likert scale. 1 = positive, 5 = negative | AB Mahmoud, D Hack-Polay, L Fuxman and M Nicoletti [17] |
|  | The coronavirus pandemic is making me feel discomfort | 5-point Likert scale. 1 = "strongly disagree," 5 = "strongly agree" |  |
|  | I feel worried about what could happen if any of my family or friends caught the virus |  |  |
| **OCB** | I do not pursue additional training to improve performance* | 5-point Likert scale. 1 = "strongly disagree," 5 = "strongly agree" | L Van Dyne, JW Graham and RM Dienesch [99] |
|  | I frequently make creative suggestions to co-workers |  |  |
|  | I produce as much as capable of at all times |  |  |
|  | Regardless of the circumstances, I deliver the highest quality work |  |  |
|  | I keep the work area clean and neat |  |  |

* Reverse-scored

**Appendix 2: Constructs’ descriptive statistics**

| **Construct** | **Whole sample (*N* = 578)** | | **Gen X (*N* = 187)** | | **Gen Y (*N* = 258)** | | **Gen Z (*N* = 133)** | |
| --- | --- | --- | --- | --- | --- | --- | --- | --- |
|  | **Mean** | **STDV** | **Mean** | **STDV** | **Mean** | **STDV** | **Mean** | **STDV** |
| **Covid-19 Perceptions** | 3.48 | 1.10 | 3.58 | 1.07 | 3.36 | 1.17 | 3.56 | 1.00 |
| **Job Insecurity** | 3.09 | 1.16 | 2.99 | 1.25 | 3.13 | 1.16 | 3.12 | 1.04 |
| **Burnout** | 3.03 | 1.19 | 2.73 | 1.13 | 3.23 | 1.20 | 3.09 | 1.16 |
| **Job Satisfaction** | 3.31 | 1.02 | 3.54 | 0.83 | 3.24 | 1.08 | 3.13 | 1.09 |
| **OCB** | 4.12 | 0.88 | 4.00 | 0.96 | 4.32 | 0.80 | 3.92 | 0.84 |
